# Anti-cancer effects of *Kaempferia parviflora* on ovarian cancer SKOV3 cells

**DOI:** 10.1186/s12906-018-2241-6

**Published:** 2018-06-11

**Authors:** Suthasinee Paramee, Siriwoot Sookkhee, Choompone Sakonwasun, Mingkwan Na Takuathung, Pitchaya Mungkornasawakul, Wutigri Nimlamool, Saranyapin Potikanond

**Affiliations:** 1 Department of Pharmacology, Faculty of Medicine, Chiang Mai University, Chiang Mai, 50200 Thailand; 2 Graduate School, Chiang Mai University, Chiang Mai, 50200 Thailand; 3 Department of Microbiology, Faculty of Medicine, Chiang Mai University, Chiang Mai, 50200 Thailand; 4 Department of Chemistry, Faculty of Science, Chiang Mai University, Chiang Mai, 50200 Thailand; 5 Environmental Science Program, Faculty of Science, Chiang Mai University, Chiang Mai, 50200 Thailand

**Keywords:** *Kaempferia parviflora*, Thai black ginger, Ovarian cancer, Anti-cancer activity

## Abstract

**Background:**

*Kaempferia parviflora* (KP) is an herb found in the north of Thailand and used as a folk medicine for improving vitality. Current reports have shown the anti-cancer activities of KP. However, the anti-cancer effects of KP on highly aggressive ovarian cancer have not been investigated. Therefore, we determined the effects of KP on cell proliferation, migration, and cell death in SKOV3 cells.

**Methods:**

Ovarian cancer cell line, SKOV3 was used to investigate the anti-cancer effect of KP extract. Cell viability, cell proliferation, MMP activity, cell migration, and invasion were measured by MTT assay, cell counting, gelatin zymography, wound healing assay, and Transwell migration and invasion assays, respectively. Cell death was determined by trypan blue exclusion test, AnnexinV/PI with flow cytometry, and nuclear staining. The level of ERK and AKT phosphorylation, and caspase-3, caspase-7, caspase-9 was investigated by western blot analysis.

**Results:**

KP extract was cytotoxic to SKOV3 cells when the concentration was increased, and this effect could still be observed even though EGF was present. Besides, the cell doubling time was significantly prolonged in the cells treated with KP. Moreover, KP strongly suppressed cell proliferation, cell migration and invasion. These consequences may be associated with the ability of KP in inhibiting the activity of MMP-2 and MMP-9 assayed by gelatin zymography. Moreover, KP at high concentrations could induce SKOV3 cell apoptosis demonstrated by AnnexinV/PI staining and flow cytometry. Consistently, nuclear labelling of cells treated with KP extract showed DNA fragmentation and deformity. The induction of caspase-3, caspase-7, and caspase-9 indicates that KP induces cell death through the intrinsic apoptotic pathway. The antitumor activities of KP might be regulated through PI3K/AKT and MAPK pathways since the phosphorylation of AKT and ERK1/2 was reduced.

**Conclusions:**

The inhibitory effects of KP in cell proliferation, cell migration and invasion together with apoptotic cell death induction in SKOV3 cells suggest that KP has a potential to be a new candidate for ovarian cancer chemotherapeutic agent.

## Background

Ovarian cancer is one of the three common gynecological cancers worldwide after cervical and uterine cancers [1]. However, it is the most leading cause of death among these three gynecologic cancers [2]. Compared to others, ovarian cancer has the poorest prognosis, with the five-year survival rate of 44% for all stages [3]. Up to 70% of all ovarian cancer cases are high-grade carcinomas which grow aggressively, metastasize rapidly, and have high chromosomal instability [4, 5]. Asymptomatic or non-specific symptoms at an early stage together with poor screening method makes ovarian cancer a late diagnostic tumor. Chemotherapeutic drugs are treatment choices for unresectable tumor. However, they have many side effects including hair loss, fatigue, bone marrow suppression, and bleeding which can lower the quality of patient life [6]. Even though many new chemotherapeutics have been developed, the drugs are less accessible for many patients due to their high cost. We hope that our findings of effective medicinal plant tested in vitro may be an important step valuable for pacing into the next level of drug discovery and to be a complementary option with reasonable cost for patients with ovarian cancer.

*Kaempferia parviflora* (KP) is a Thai traditional plant in the Zingiberaceae family. It is commonly known as Thai black ginger or in Thai as “Krachai dum”. KP has been previously demonstrated to have several pharmacological effects including anti-plasmodial, anti-fungal, anti-mycobacterial [7], and anti-cancer properties [7–9]. We previously described the anti-cancer property of KP against cervical cancer HeLa cells showing the promising possibility that KP may be used as a potential agent for cervical cancer treatment [10]. However, the anti-cancer effects of KP against ovarian cancer have not yet been reported. This leads us to investigate anti-cancer properties of KP against a high-grade ovarian cancer cell line, SKOV3, which is highly resistant to many cytotoxic agents. Since epidermal growth factor receptor (EGFR), is strongly expressed in ovarian cancer [11] and involved in cell proliferation, cell migration, cell survival, and metastasis, we therefore examined the effects of KP on SKOV3 alone and under the influence of EGF to verify whether KP can overcome the EGF-dependent growth and survival signal transduction pathways. Nevertheless, the molecular mechanisms of how KP suppresses tumor growth and survival were also explored. In particular, the effects of KP on the PI3K/AKT and MAPK pathways which are important signal transduction pathways for tumorigenesis [12, 13] were defined.

## Methods

### Cell culture

Human ovarian cancer SKOV3 cells were obtained from ATCC (ATCC, Manassas, VA, United States) and maintained in (Roswell Park Memorial Institute) RPMI-1640 medium (Gibco, BRL, USA) supplemented with 10% fetal bovine serum (FBS) (Gibco BRL, USA) and antibiotics (100 U/mL penicillin and 100 μg/mL streptomycin) (Caisson, USA) and incubated at 37 °C in a humidified atmosphere, 5% CO_2_. The cells were sub-cultured every 2–3 days.

### Extraction of *Kaempferia parviflora* rhizomes

The rhizomes of *Kaempferia parviflora* with voucher specimen number (R-CMUKP002) authenticated by Dr. Angkhana Inta and deposited at the Faculty of Science, Chiang Mai University, Thailand, were harvested from the CMU-RSPG Kaempferia housing at Chiang Dao, Chiang Mai Province, Thailand. For the extraction, chopped rhizomes of the plant were extracted with 95% ethanol at room temperature (RT) for 3 days and filtered before concentrated using a rotary evaporator. After solvent evaporation, the plant ethanolic extraction yielded 9.85% dry weight of KP rhizomes. One milliliter of DMSO was used to dissolve 1 g of KP extract to make a 1 g/mL stock solution. The KP stock was pre-diluted in medium prior to each treatment. Each experiment was performed with three independent batches of KP extract, each assayed in triplicate. The final concentration of DMSO was maintained below 0.5% *v*/v throughout the experiment.

### Cell viability assay

The cytotoxicity of KP on SKOV3 cells was determined by MTT (3-(4, 5-dimethylthiazol-2-yl)-2, 5-diphenyl tetrazolium bromide). Cells were seeded at a density of 1 × 10^4^ cells per well in 96-well plates overnight and treated with KP or DMSO (vehicle control 0.006–0.1%) in quadruplicate. For the treatment group, cells were incubated with complete media containing different concentrations of KP extract, ranging from 0 to 10 mg/mL with or without the presence of 100 ng/ml of EGF. After 24 h, cells were incubated with 0.5 mg/mL MTT reagent (Applichem GmbH, Germany) for 1–3 h. The culture supernatant was aspirated and 100 μl of DMSO was added to each well. The absorbance was measured at 570 nm using Synergy™ H4 Hybrid Multi-Mode Microplate Reader. Cell viability assay was performed in 3 individual experiments.

### Cell counting

Cells were seeded in 24-well plates at a density of 0.05 × 10^6^ cells/well in culture media and incubated for 24 h at 37 °C, 5% CO_2_. Cells were treated with KP extract at non-toxic concentrations (0.01, 0.025, and 0.05 mg/mL). The total number of cells at different time points (0, 24, 48, 72 and 96 h) was counted using a haemacytometer. The doubling time of the cell was calculated according to the following formula: Doubling time = (Time×log2)/(log(final number)-log(initial number)).

### Gelatin zymography

The activity of MMP-2 and MMP-9 was examined using gelatin zymography. The sample culture supernatants of SKOV3 cells (1 × 10^6^ cells in a 3-cm dish) incubated with different concentrations of KP extract (0, 0.01, 0.05, and 0.1 mg/mL) with or without the presence of EGF (100 ng/mL) for 24 h were collected. The sample culture supernatants were separated in 10% sodium dodecyl sulfate polyacrylamide gel electrophoresis (SDS-PAGE) containing 0.1 mg/mL of gelatin B (Bio-Rad Laboratories, Hercules, California, USA) under a non-reducing condition in cold running conditions. After electrophoresis, the gels were incubated with 2.5% Triton X-100 twice (for 30 min each), at RT and washed with 10 mM Tris buffer, pH 8.0 for 2 min. The gels were incubated with 1% gelatinase buffer (50 mM Tris HCl, 10 mM CaCl_2_, pH 8) overnight at 37 °C. The gels were stained with 0.5% (w/v)  Coomassie brilliant blue R250 (Bio-Rad Laboratories) in 50% methanol and 10% glacial acetic acid for 30 min and destained with a destaining solution (10% acetic acid and 50% methanol). Proteolytic activities of MMP-2 and MMP-9 were visualized as clear zone bands on a blue background and analyzed using ImageJ software.

### Wound healing assay

SKOV3 cells (0.5 × 10^6^ cells/well) were seeded and cultured in 24-well plates for 24 h. A scratch wound was made by using 200 mL pipette tip. Cells were treated with different concentrations of KP extract (0.01, 0.05, and 0.1 mg/mL) for 24 h. Images of the scratched wounds were captured at different time points (0, 12, and 24 h). The closing of scratched wounds was considered to be the completion of the migration process. The migrated areas were analyzed and determined using the ImageJ software.

### Cell migration

A Cell Culture Insert (8 μm) (SPL Life Sciences, South Korea) was used to confirm the effect of KP on suppressing cell migration. Cells at a density of 0.3 × 10^6^ cells/well were seeded in the upper chambers and cultured in serum-free media for 24 h. The next day cells in the upper chamber were treated with different concentrations (0, 0.01, 0.05, and 0.1 mg/mL) of KP in serum free media (SFM), and the upper chambers were put into the (lower) wells containing RPMI with 5% FBS and incubated for 24 h. Absolute methanol was used to fix cells for 5 min at RT, and cells were then stained with 0.5% crystal violet for 30 min. The upper chambers were washed for 3 times with water, and cells attached to the surface inside the chamber were removed with a cotton swab and the stained cells attached at the other site of the chamber were captured and analyzed with the ImageJ software.

### Cell invasion assay

The effects of KP on SKOV3 cell invasion were determined using Cell Culture Inserts (SPL life sciences, Korea). The polycarbonate invasion chambers (8 μm pore size) were coated with Matrigel® Matrix (356,234, Lot 4,272,006, Corning, Bedford, USA) per well and incubated at RT for 1–4 h. Cells, at a density of 0.25 × 10^6^ cells per well, were seeded on Matrigel with 0.01 and 0.05 mg/mL of KP in serum-free media and the invasion chambers were put into the wells (the lower) containing RPMI with 10% FBS and incubated for 20 h. Cells were then fixed with absolute methanol for 5 min at RT and stained with 0.5% crystal violet for 15 min. After three washes with water, cells in the invasion chambers were removed with cotton swab and the pictures of the stained cells attached at the other site of the invasion chamber were taken and analyzed with ImageJ software.

### Trypan blue exclusion test

Cells were seeded at a density of 0.05 × 10^6^ cells/well in 24-well plates and incubated with different cytotoxic concentrations (0, 0.05, 0.1, and 0.25 mg/mL) of KP extract. Cells were harvested after 3, 6, 12, 24, and 48 h of incubation. Trypan blue solution (Gibco, USA) was added to the cell suspensions in a ratio of 1:1. Total cells and dead cells (stained in blue) were counted using haemacytometer. The percentage of living cells and dead cells was calculated.

### Cell apoptosis assay

Cell apoptosis was assessed by annexin-V-FITC/propidium iodide (PI) staining. Cells were seeded at a 0.3 × 10^6^ cells/well density in 3-cm cell culture dishes and cultured for 24 h. Cells were treated with different concentrations of KP extract (0, 0.1, 0.3, and 0.5 mg/mL) for 12 h. Cells were harvested and resuspended in 1X annexin-V binding buffer (50 mM Tris-HCl (pH 7.5), 5 mM EDTA, 0.5 mM DTT, 50% glycerol). Cells were incubated with annexin V-FITC (ImmunoTools, Germany) and propidium iodide (PI) (Sigma Aldrich) for 15 min in the dark at RT before performing flow cytometry.

### Nuclear staining

SKOV3 cells were seeded at a density of 0.5 × 10^6^ cells/well on glass coverslips for 24 h. Cells were treated with KP extract at different concentrations (0.1, 0.3, and 0.5 mg/mL) and incubated for 7 h. Cells were fixed with 4% paraformaldehyde/PBS at RT for 15 min. Then, cells were washed thrice and incubated with 5 μg/mL of Hoechst 33342  in PBS (Thermo Fisher Scientific. Thailand) for 1 h. Following staining, the sample slides were washed twice with PBS for 5 min each time, and the sample slides were mounted using Fluoromount media (SouthernBiotech, United States). Cells were observed by a fluorescent microscope, AX70 Olympus R, Japan, with 40X magnification, and micrographs were captured with the DP-BSW Basic Software for the DP71 microscope digital camera.

### Western blot analysis

SKOV3 cells were seeded in 3-cm dishes at a density of 0.3 × 10^6^ cells/well for 24 h. The next day, media were changed to SFM and cells were cultured for 24 h. Cells were treated with KP extract at non-toxic concentrations (0.01 and 0.05 mg/mL) for 6 h and 100 ng/mL of EGF was added to the wells 15 min before harvesting cells. Cell lysates were prepared by adding 300 μL of 1X reducing Laemmli buffer and heating at 95 °C for 5 min. Cell lysates were separated by SDS-PAGE for 90 min at 140 V and transferred to PVDF membranes (Immobilon-P; Millipore, Bedford, MA) for 120 min at 100 V. After electrophoresis, membranes were blocked with 5% skim milk in TBS containing 0.1% tween-20 (TBST) at RT for 1 h. The blots were incubated with primary antibodies (1:10000 of anti-β-actin, 1:5000 of anti-pERK1/2, 1:5000 of anti-pAKT, 1:5000 of anti-ERK1/2, 1:5000 of anti-AKT, 1:3000 of caspase-3, or caspase-7, or caspase-9) at 4 °C overnight. Anti-β-actin was obtained from US biological (USA) and the remaining antibodies were purchased from Cell Signaling Technology (USA). The membranes were washed and incubated with an anti-mouse Ig conjugated with IRDye®800CW (1:5000) or an anti-rabbit Ig conjugated with IRDye®680RT (1:5000) at RT for 2 h. The immunoreactive bands were visualized by Odyssey ® CLx Imaging System - LI-COR Biosciences (USA). The bands were analyzed using Image Studio Lite.

### Statistical analysis

Data are presented as mean ± SD. Data were analyzed by one-way ANOVA and *P*-value < 0.05 was considered statically significant.

## Results

### The effect of KP on SKOV3 cell viability and cell proliferation

**Fig. 1:**
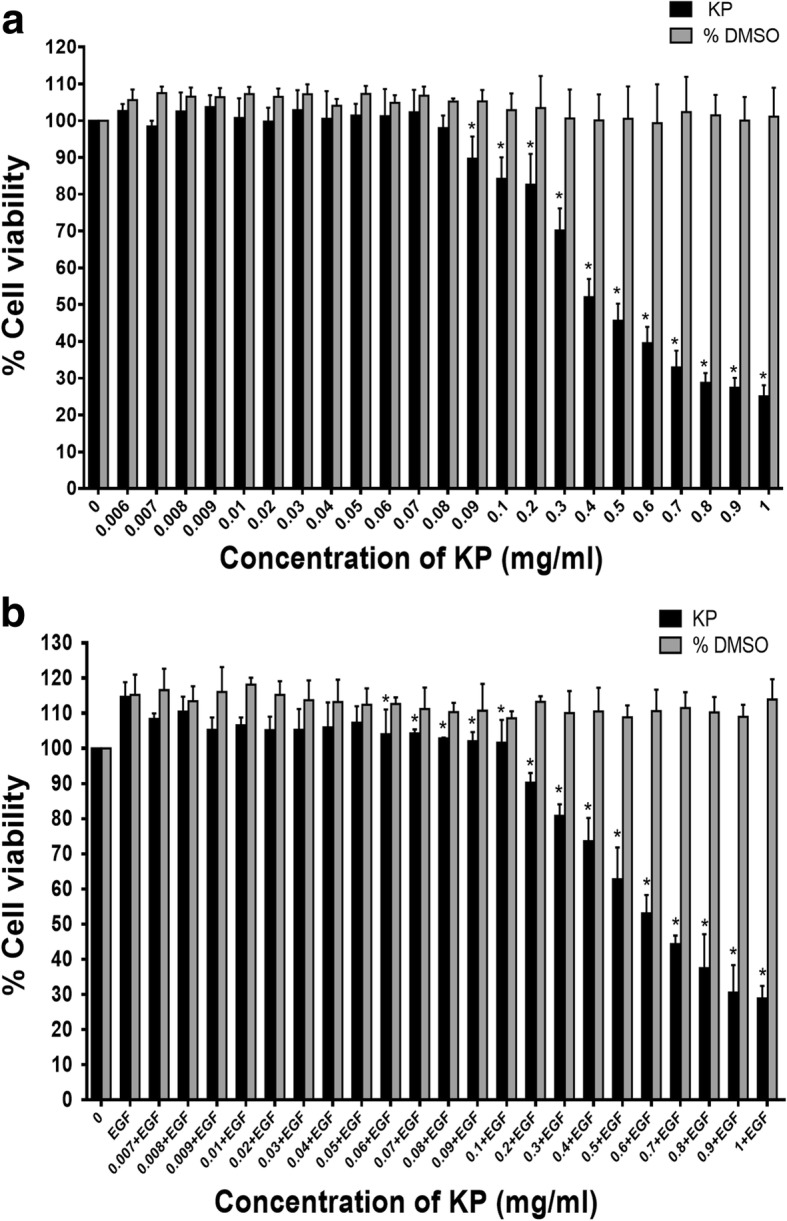
The cytotoxicity effect of different concentrations of KP ethanol extract on SKOV3 cells without EGF (**a**) and with 100 ng/mL EGF (**b**). All data were from 3 independent experiments and reported as means ± SD of each quadruplicate ^*^P < 0.05 compared to the control (untreated and EGF)

**Fig. 2:**
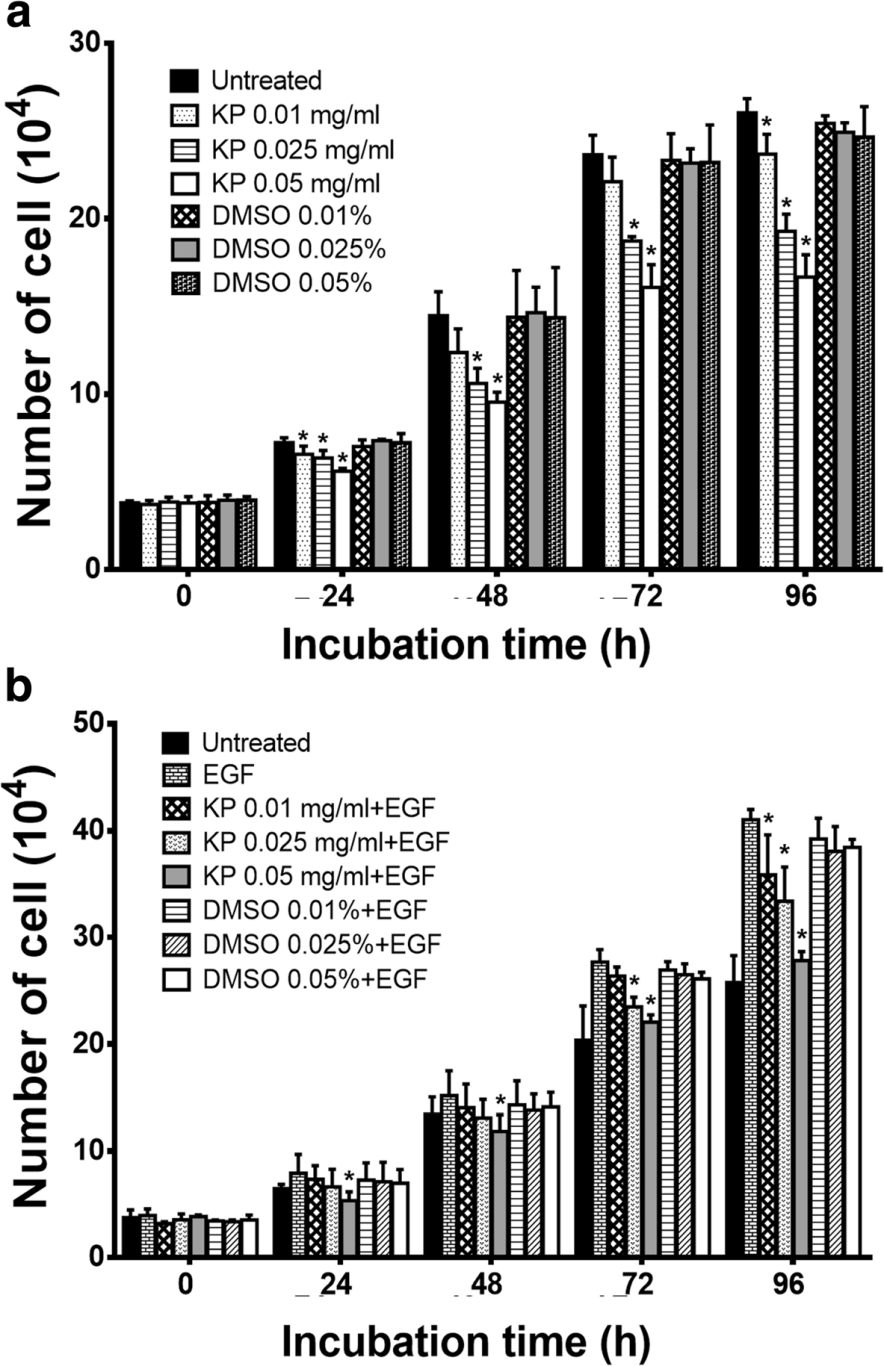
The number of SKOV3 cells treated with non-toxic concentrations of KP (0.01, 0.025, 0.05 mg/mL) without EGF (**a**) and with 100 ng/mL EGF (**b**) at 24, 48, 72 and 96 h. Data are expressed as mean ± SD (*n* = 3). ^*^P < 0.05 as compared to untreated (**a**) or EGF (**b**)

To investigate antitumor properties of KP, we first evaluated its cytotoxicity to SKOV3 by using MTT assay. We found that cells treated with KP extract at different concentrations (0.006–1 mg/mL) for 24 h showed significant reduction in cell viability in a concentration-dependent manner from the range of 0.09 mg/mL to 1 mg/mL as shown in Fig. 1a. The half maximal inhibitory concentration (IC50) of KP extract was 0.53 ± 0.08 mg/mL. Since epidermal growth factor receptor (EGFR), which is highly expressed in ovarian cancer cells, is a very important factor for tumor growth [11], we therefore stimulated SKOV3 cells with EGF and performed MTT assay to evaluate whether KP still be able to suppress cell viability. As shown in Fig. 1b, EGF significantly increased cell viability approximately 15%. Interestingly, in the presence of EGF, KP still exhibited strong growth suppression in a concentration-dependent manner. The vehicle control, DMSO, at all concentrations, did not show any cytotoxic effect. The IC50 of KP extract in the presence of EGF was 0.63 ± 0.08 mg/mL which is similar to the IC50 of KP treatment without EGF. We further performed cell counting at 24, 48, 72, 96 h after KP treatment and found that KP extract significantly reduced the number of cells in a concentration-dependent manner (Fig. 2). These observations were still seen in the treatment with the presence of EGF. The number of cell from different time points were used to calculate the doubling time which is the time required for cell dividing from one to two cells. The doubling time of SKOV3 cell was approximately 24 h. Interestingly, cells treated with KP at 0.025 and 0.05 mg/mL significantly increased cell doubling time to 32.6 h, and 31.5 h, respectively.

### The effect of KP on inhibiting MMP-9 and MMP-2 activities

**Fig. 3:**
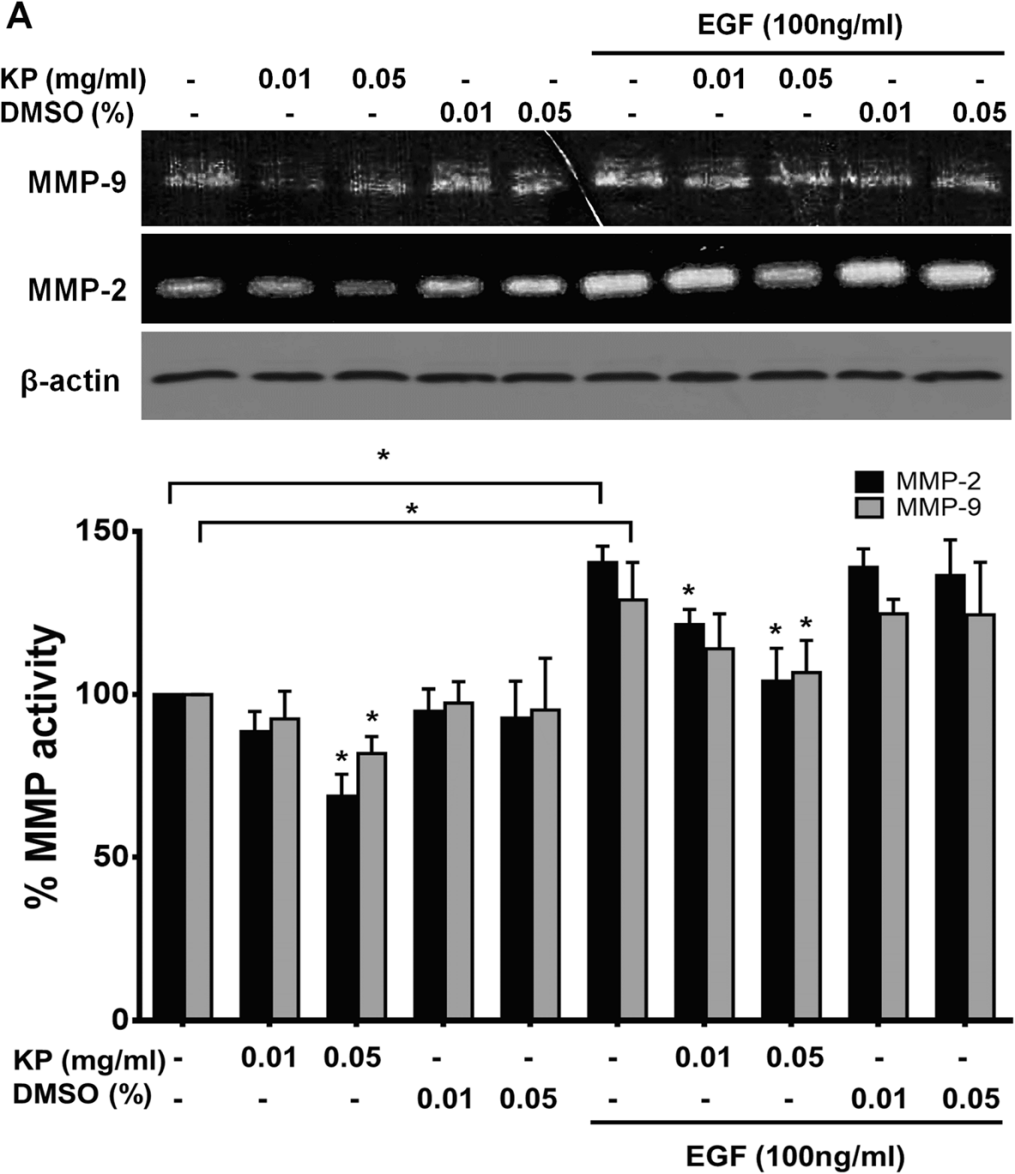
The effect of KP ethanol extract on MMP-9 and MMP-2 activity. Gelatin zymogram showing MMP9 and MMP2 activities (**a**), Immunoreactive bands of β-actin was used as a loading control. Histogram of MMP-2 and MMP-9 activity is presented as percent of activity (**b**). All data were from 3 independent experiments. ^*^P < 0.05

We next determined whether KP extract suppresses MMP-9 and MMP-2 activities. Data from zymographic analysis showed that cells treated with KP extract at 0.01 and 0.05 mg/mL reduced MMP-9 activity to 92.52 ± 8.55% and 81.92 ± 5.18% and MMP-2 activity to 88.66 ± 6.17 and 68.83 ± 6.17%, respectively (Fig. 3a and b). EGF at 100 ng/mL strongly increased MMP-2 and MMP-9 activities over 140%. As we expected, KP extract with the presence of EGF was still able to suppress MMP-9 and MMP-2 activities. The percent reduction of MMP-9 activity was 113.97 ± 10.7 and 106.64 ± 9.9 mg/mL and MMP-2 activity was 121.4 ± 4.7and 104.01 ± 10.12 mg/mL for cell treated with KP at 0.01 and 0.05 mg/mL, respectively. The immunoreactive bands of β-actin detected by western indicated the equal amount of cells in all treated groups.

### The effect of KP on inhibiting cell migration and invasion

**Fig. 4:**
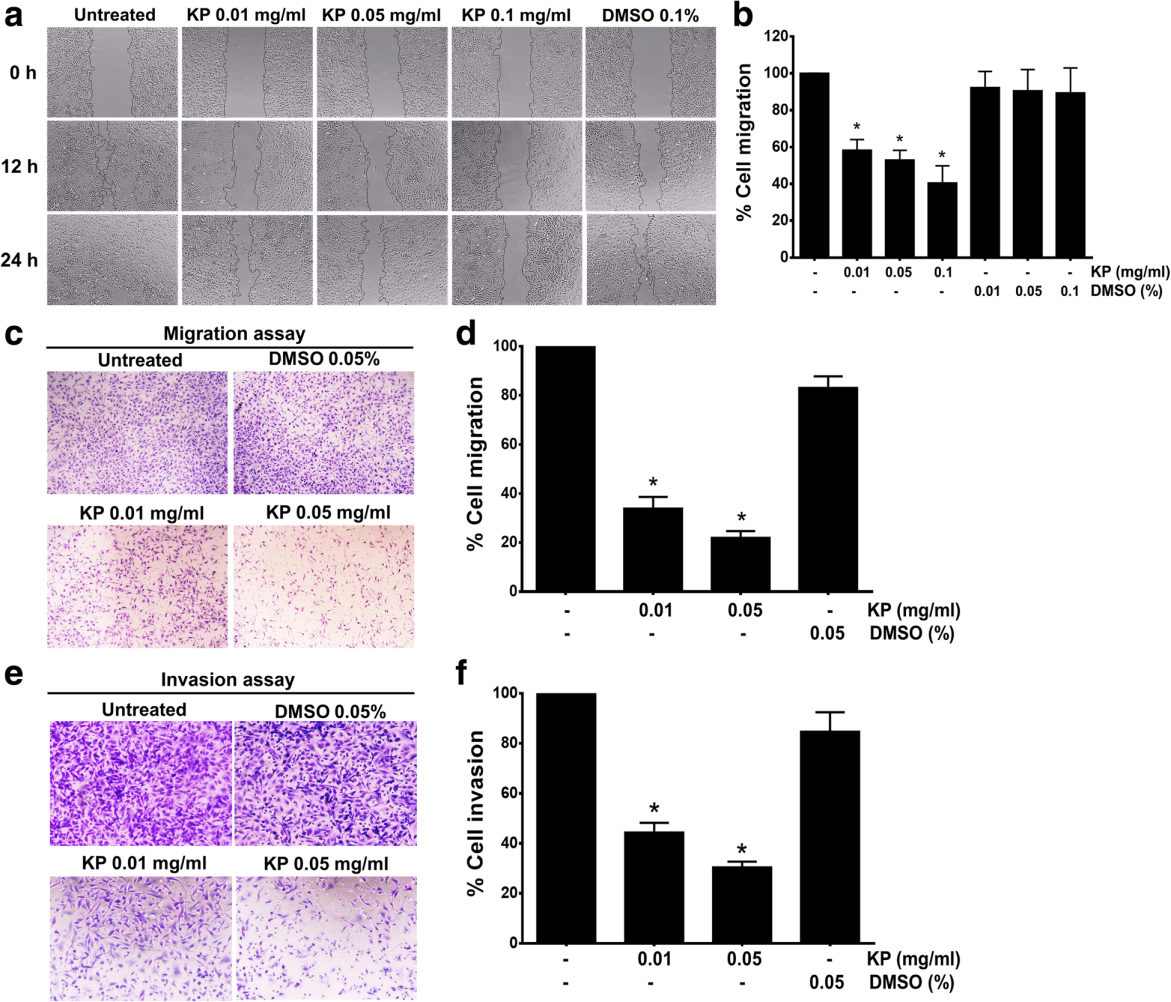
The effect of KP on SKOV3 cell migration. Wound-healing assay of SKOV3 cells treated with KP ethanol extract at 0, 12, and 24 h after performing the scratch (**a**). Histogram represents the percentage of cell migration (**b**). Transwell migration assay and represented histogram are shown in **c** and **d**. Invasion assay was shown in **e** and **f**. All data were from 3 independent experiments and reported as means ± SD for measurements in quadruplicate ^*^P < 0.05 as compared to the control

Based on the fact that MMP-9 and MMP-2 are crucial factors for tumor migration and metastasis, we therefore performed wound healing assay to examine cell migration and found that cells treated with KP at 0.01, 0.05, and 0.1 mg/mL effectively reduced the percent of cell migration to 58.30 ± 5.8, 52.91 ± 5.32, and 40.50 ± 9.27%, respectively (Fig. 4a and b). Moreover, we confirmed the ability of KP in inhibiting cell migratory function of SKOV3 cells with Transwell migration assay. The results showed that SKOV3 cells without any treatment could migrate through the upper well to the lower chamber. However, the number of migrated cells was drastically decreased in cells treated with KP extract whereas the vehicle control did not show any inhibitory effect on SKOV3 cell migration (Fig. 4c and d). The effect of KP on cell invasion was determined by using Transwell assay with the presence of matrigel. Similar to cell migration assay, cells treated with KP 0.01 and 0.05 mg/mL showed a decrease in percent cell invasion to 44.42 ± 2.37%, and 30.46 ± 2.23%, respectively (Fig. 4e and f) while the DMSO vehicle did not show significant inhibitory effect on cell invasion.

### The effect of KP on inhibiting growth and survival signal transduction pathways

**Fig. 5:**
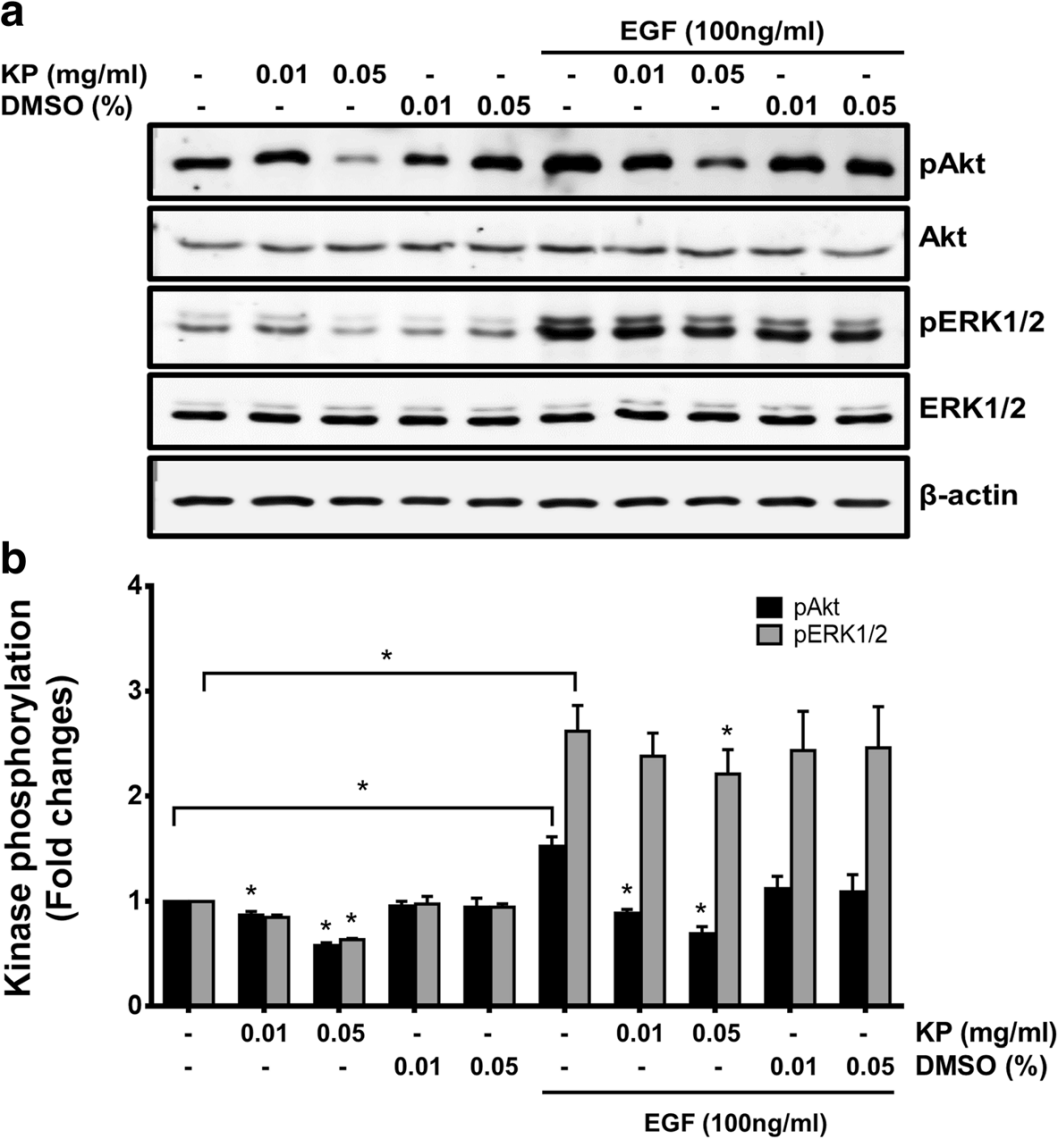
The effect of KP on the PI3K/AKT and EKR1/2 MAPK signal transduction in SKOV3 cells. The immunoreactive bands of pAKT, AKT, pERK1/2 and ERK1/2 (**a**). Histogram of phosphorylation level of AKT and ERK1/2 (**b**). β-actin was used as a loading control. Data expressed as mean ± SD (*n* = 3). ^*^P < 0.05

Several signaling molecules are involved in cell growth and survival processes in response to EGF stimulation. Those molecules include ERK1/2 and AKT  proteins. We therefore investigated the possible underlying mechanism of KP that suppresses growth in SKOV3 cells. As shown in Fig. 5,  we found that cells treated with KP at 0.01 and 0.05 mg/mL exhibited reduction in ERK1/2 phosphorylation to 0.85 ± 0.02 and 0.64 ± 0.031, respectively. Even though EGF strongly activated ERK1/2 phosphorylation (2.6 fold), KP at 0.01 and 0.05 mg/mL was able to reduce the phosphorylation of ERK1/2 to 2.38 ± 0.22 and 2.21 ± 0.23, respectively. Moreover, KP extract at 0.01 and 0.05 mg/mL reduced the phosphorylation of AKTto 0.87 ± 0.04 and 0.58 ± 0.03 without the presence of EGF and to 0.89 ± 0.04 and 0.7 ± 0.07 with the presence of EGF, respectively.

### The effect of KP on inducing apoptotic cell death

**Fig. 6:**
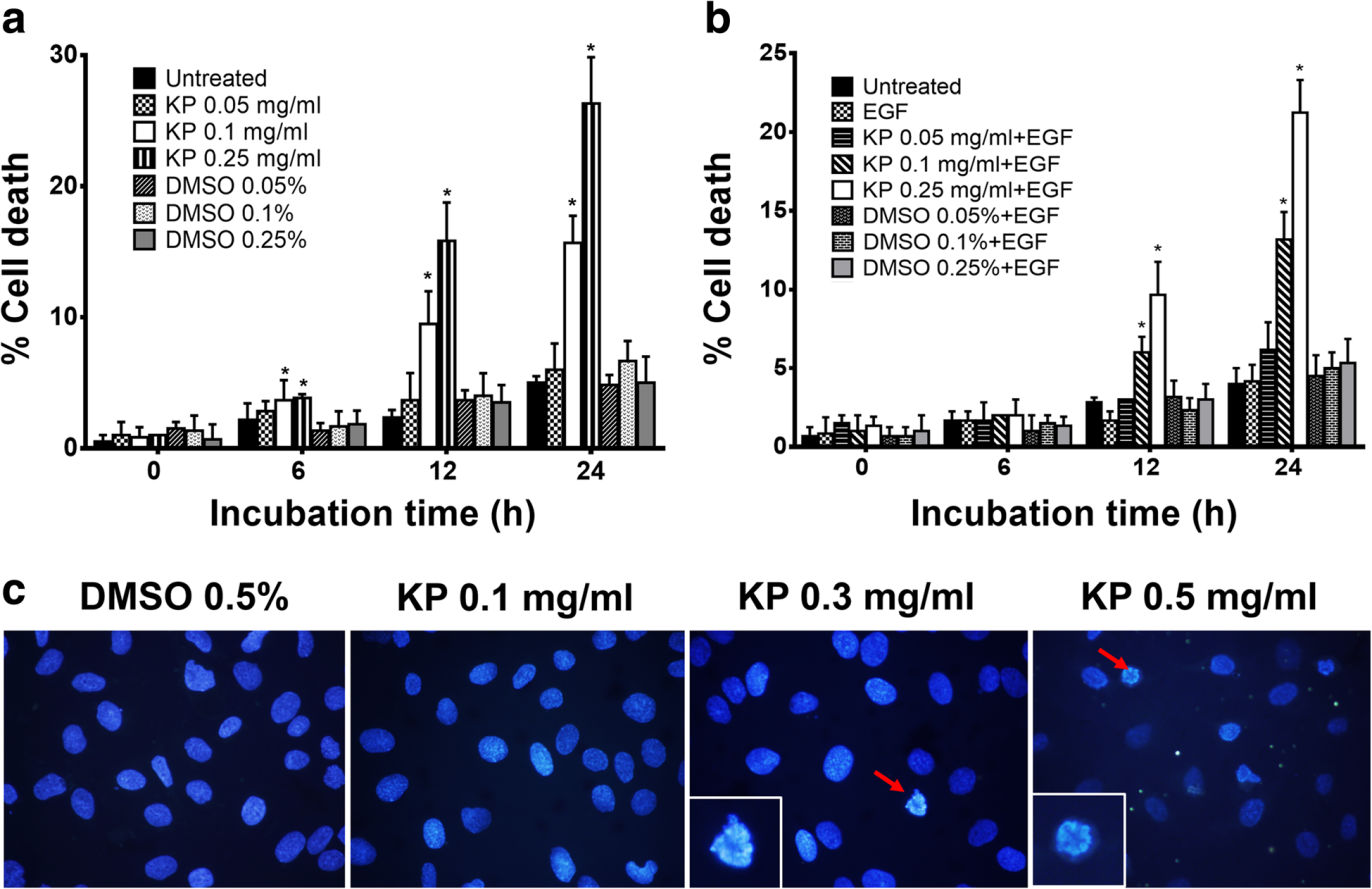
The effect of KP on cell death in SKOV3 cells by trypan blue exclusion assay. Percent of cell death of cells treated with KP without EGF (**a**) and with 100 ng/mL EGF (**b**). Data presented as means ± SD, *n* = 3, ^*^P < 0.05 compared to DMSO control. The DNA staining (Hoechst 33342) of KP treated cells shows nuclear deformity (**c**). The condensation of the nucleus was observed in KP ethanol extract treatments compared to the vehicle control DMSO. Original magnification, 400X

**Fig. 7:**
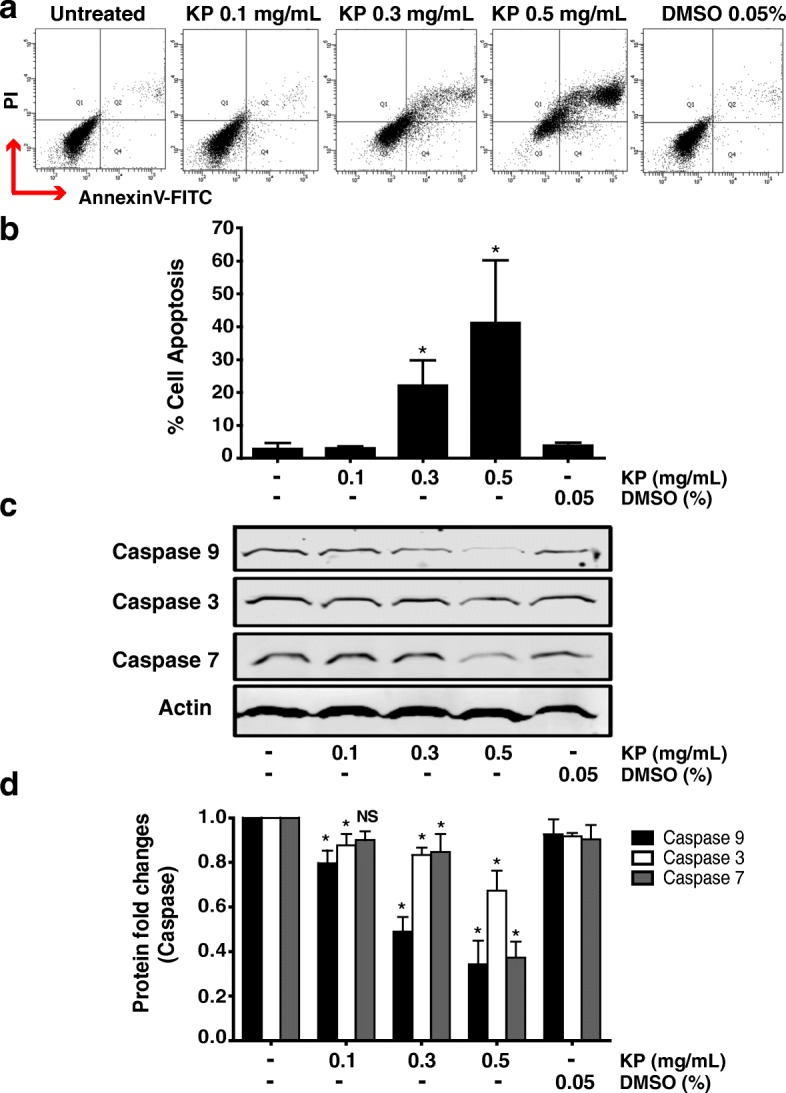
The effect of KP ethanol extract on apoptosis and caspases in SKOV3 cells. Annexin V-FITC and PI labeling in KP treated cells was measured by flow cytometer (**a**) and histogram of percent of apoptotic cells is shown in **b**. The level of caspase-3, - 9, and  -7 by western blotting are shown in **c**. Histogram of relative intensity of full-length of caspase-3, − -9, and -7. β-actin is used as a protein loading control (**d**). These data are represented as mean ± SD of three replicates. ^*^P < 0.05 indicates significant difference compared to control

Since cell viability assay showed cytotoxicity of KP extract at the concentrations over 0.1 mg/mL. We thus examined whether KP extract increases cell death by using trypan blue exclusion test. We found that cells treated with KP extract at 0.1 and 0.25 mg/mL significantly increased cell death after 24 h of incubation. The percentage of cell death was 15.67 ± 2% and 26.33 ± 3.5% with KP treatment at 0.1 and 0.25 mg/mL, respectively (Fig. 6a). Importantly, with the presence of EGF 100 ng/mL, KP extract at 0.1 and 0.25 mg/mL was able to induce cell death to 13.17 ± 1.8% and 21.25 ± 2%. In order to confirm whether dead cells were apoptotic cell, fluorescence nuclear staining using Hoechst 33342 was performed after treating cells with KP for 6 h. Figure 6c showed nuclear fragmentation of SKOV3 cells treated with KP extract at 0.3 and 0.5 mg/mL. This effect was seen in cells treated with DMSO. Based on this observation, we speculated that KP extract induces cell death via apoptosis machinery. We next determined apoptotic event by performing AnnexinV  and PI fluorescent staining and detecting with flow cytometer. We found that cells treated with KP extract at 0.3 and 0.5 mg/mL increased apoptosis to 22.13 ± 7.6% and 41.13 ± 19.15%, respectively (Fig. 7a, b). We further examined the activation of caspase-9, caspase-3, and caspase-7 by western blot analysis and found that the full length of all caspases was significantly reduced in a concentration-dependent manner (Fig. 7c, d). These data strongly suggest that KP induces cell death via the activation of apoptotic cell death.

## Discussion

Ovarian cancer is the most common cause of cancer death among other gynecologic cancers [14]. No obvious symptoms are present at the early stage, thus, most of patients are diagnosed when cancer is in an advanced stage which gives rise to poor response to chemotherapies [15]. Staging is an important factor determining prognosis and clinical outcome. Stage I, is defined as tumor is confined in the ovary. This stage shows overall survival of approximately 84 months. In particular, stage IV has much less overall survival rate of only 10 months [15]. The survival rate is not only related to stages of the disease but also associated with ovarian cell types. Several cell types have been identified in ovarian cancer. The most common histologic subtype is high-grade serous adenocarcinoma which has the worst prognosis [16, 17]. We are particularly interested in using a high-grade serous adenocarcinoma, SKOV3, cell line as a model for investigating anticancer activity of *Kaempferia parviflora* in ovarian cancer.

We first performed cell viability assay to evaluate the cytotoxicity of KP extract in SKOV3 cells. It was found that KP extract decreased cell viability of ovarian cancer in a concentration-dependent manner with IC50 of approximately 0.5 mg/mL, which was slightly higher than the previously reported IC50 of KP in cervical cancer cell line, HeLa [10]. Interestingly, KP extract also strongly exhibited the reduction of ovarian cancer cell viability in the presence of EGF, suggesting that KP has potent cytotoxic effects, which overcome the influence of EGF in maintaining cell viability. We further discovered that treating cells with KP extract at non-toxic concentrations (with or without the presence of EGF) at various time points could significantly inhibit number of cells in a concentration-dependent manner. Therefore, the doubling time was increased to approximately 1.3–1.4 folds compared to untreated SKOV3 cells. This observation suggests that KP extract may suppress SKOV3 cell proliferation. According to cell viability results, the non-toxic concentrations were chosen for further experiments.

Since SKOV3 cells are known to be a high grade serous adenocarcinoma which has been reported to have high metastatic rate [16, 18]. One major factor that plays important roles in cell invasion and metastasis is matrix metalloproteinase (MMP). Extensive evidence has been shown that the increased MMP level correlates with tumor progression and metastasis, especially in advanced ovarian serous cancers [19, 20]. MMP expression, particularly MMP-2 and MMP-9, has been shown to have clinical association with progression of ovarian cancer [21, 22]. MMPs degrade various components of the extracellular matrix and play a crucial role in tumorigenesis, migration, invasion, and metastasis [23], and inhibition of MMPs by specific inhibitors has been demonstrated to markedly suppress tumor invasion and metastasis [24, 25]. Based on these previous reports, we hypothesized that KP extract may be able to modulate the expression of MMPs. Undoubtedly, our zymographic study revealed that KP extract dramatically inhibited the activity of MMP-2 and MMP-9 in a concentration-dependent manner in SKOV3 cells. The ability of KP extract in suppressing the activity of MMPs was independent on the presence of EGF, since KP extract could be able to strongly overcome the effects of EGF. Our findings are in line with our previous studies showing that KP suppressed MMP-2 production in cervical cancer, HeLa cells [10]. Similar observation was reported in colorectal carcinoma cells, where a flavonoid, myricetin, inhibited MMP-2 activity and cell invasion [26]. These let us to believe that KP extract may also be able to reduce cancer cell migration and invasion. We, therefore, further investigated the effects of KP extract on cell migration and invasion.

Generally, cancer cell migration is involved in altering the cell-matrix interface on the cell surface [27]. The overexpression of MMPs could enhance cell migration [28], whereas the inhibition of MMP activity or overexpression of tissue inhibitor of metalloproteinases (TIMPs) resulted in a decrease in cancer cell migration [29]. Our results from wound healing assays showed that KP extract suppressed cell migration of SKOV3 cells in a concentration-dependent manner. Furthermore, the migratory function of cells was confirmed by Transwell migration showing that KP extract drastically inhibited migration and invasion of SKOV3 cells. The results from invasion assay with the presence of matrigel definitively verified that KP extract could be able to suppress invasion of SKOV3 cells. Together, these results strongly suggest that KP possesses the inhibitory effect on migration and invasion of SKOV3. These observations are consistent with the zymography results, suggesting that the reduced activity of MMPs may greatly contribute to the reduction of cancer cell invasion and migration.

Besides the ability of KP extract on an aspect of suppressing ovarian cancer cell metastasis, we would also like to explore its effect on ovarian cancer cell growth and survival. In particular, since SKOV3 cells apparently express EGF receptor (EGFR) [30], we therefore examined whether KP extract can overcome the influence of EGF on activating molecular signal transduction pathways relevant to cell growth and survival. EGFR is involved in cell proliferation, motility, adhesion, angiogenesis, and survival via the activation of phosphatidylinositol-3 kinase (PI3K/AKT) pathway, and the extracellular signal-regulated kinase (ERK) pathway [31]. EGFR is widely expressed in 33–75% of ovarian cancer and has been implicated in the growth and progression of this cancer [32–34]; therefore, EGFR is important to represent a potential target for anticancer drug development. An example of EGFR-directed monoclonal antibody is cetuximab, which inhibits cell growth in OVCAR-2 cells, whereas the growth of SKOV3 cells is not affected [35]. Another class of EGFR inhibitor is a group of small molecule tyrosine kinase inhibitors that target the receptor catalytic domain of EGFR. Those include gefitinib and erlotinib [36]. AKT and ERK are major downstream signaling molecules of EGFR [37]. Previous evidence demonstrated that KP extract significantly suppressed the phosphorylation of PI3K, AKT, ERK1/2, and Elk1 in HeLa cells [10], thus we hypothesized whether KP extract can suppress the activation of ERK1/2 and AKT signaling in high grade serous ovarian cancers. Our study clearly showed that KP extract markedly suppressed phosphorylation of ERK1/2 and AKT. This observation was still seen when the experiment was performed with the presence of EGF. The present results indicate that KP extract suppresses ERK1/2 pathway which is normally involved in cell proliferation, and the extract suppressed AKT pathway which plays roles in cell survival. A study in *Drosophila* showed that a gain-of-function mutation that results in enhanced ERK1/2 signaling capabilities could support ERK1/2 activation in the cancer cells [38]. Our findings are supported by recent studies showing that the use of RNA interference to silence ERK1/2 phosphorylation led to the complete suppression of tumor cell proliferation [39]. Since various cancers have aberrant regulation of AKT pathway that leads to prolonged survival of tumor [40], and previous studies showed that the inhibition of AKT activity is useful as a therapeutic approach for the therapy of cisplatin-resistant ovarian cancer because an activation of AKT promotes cisplatin-resistance [41, 42], we hope that KP may be a novel and effective agent that has some potential targets in AKT signaling and therefore may be beneficial for developing a cancer therapeutic means.

One of our key observations was an increase in cell death at 24 h after treatment with KP extract at toxic concentrations. Nuclear fragmentation, which is a result of the cleavage of chromosomal DNA into oligonucleosomal size fragments, is an integral part of apoptosis [43]. The cell apoptosis leads to deformity of nuclear lamina, and consequently increases active caspases [44]. Therefore, to confirm whether these dead cells were apoptotic cells, we stained SKOV3 cells with Hoechst 33342 and observed nuclear fragmentation of SKOV3 cells treated with KP extract. This finding is consistent with Potikanond et al. (2017) indicating that nuclear deformity and nuclear fragmentation were induced by KP treatment in HeLa cells. In addition, the apoptotic event of KP-treated SKOV3 was further determined by Annexin V/PI and flow cytometry analysis. Clearly, our results showed that KP significantly induced apoptosis in SKOV3 cells in a concentration-dependent manner. To confirm our hypothesis that KP extract induces apoptosis in ovarian cancer cells, we specifically analyzed the key apoptosis execution enzymes, caspase-3, caspase-7, and caspase-9 in SKOV3 cells treated with KP extract. The results showed that the full-length structure of all caspases was reduced in a concentration-dependent manner in SKOV3 cells treated with KP extract, implicating that SKOV3 cell death in KP treatment was possessed through programed cell death signaling pathway.

## Conclusions

The current study demonstrated that KP extract has anti-cancer properties against a high grade serous adenocarcinoma, SKOV3. Specifically, even though SKOV3 cells are every aggressive and resistant to many chemotherapeutic agents, our results showed that KP extract was able to suppress the activity of MMP-2 and MMP-9, migration and invasion, activation of growth and survival signal transduction pathways, and induction of apoptotic cell death. These observations convince us to believe that KP extract is a potential agent to be further developed as an effective therapy for ovarian cancer.

## Abbreviations

DMSO: Dimethyl sulfoxide
EGF: Epidermal growth factor
KP: *Kaempferia parviflora*
MMP: Matrix metalloproteinase
